# Radiological features of experimental staphylococcal septic arthritis by micro computed tomography scan

**DOI:** 10.1371/journal.pone.0171222

**Published:** 2017-02-02

**Authors:** Farah Fatima, Ying Fei, Abukar Ali, Majd Mohammad, Malin C. Erlandsson, Maria I. Bokarewa, Muhammad Nawaz, Hadi Valadi, Manli Na, Tao Jin

**Affiliations:** 1 Department of Rheumatology and Inflammation Research, Institute of Medicine, Sahlgrenska Academy at University of Gothenburg, Göteborg, Sweden; 2 Department of Pathology and Forensic Medicine, Ribeirao Preto School of Medicine, University of Sao Paulo, Sao Paulo, Brazil; 3 Department of Microbiology and Immunology, Affiliated Hospital of GuiZhou Medical University, Guiyang, P.R. China; 4 Department of Rheumatology, Sahlgrenska University Hospital, Göteborg, Sweden; US Department of Veterans Affairs, UNITED STATES

## Abstract

**Background:**

Permanent joint dysfunction due to bone destruction occurs in up to 50% of patients with septic arthritis. Recently, imaging technologies such as micro computed tomography (μCT) scan have been widely used for preclinical models of autoimmune joint disorders. However, the radiological features of septic arthritis in mice are still largely unknown.

**Methods:**

NMRI mice were intravenously or intra-articularly inoculated with *S*. *aureus* Newman or LS-1 strain. The radiological and clinical signs of septic arthritis were followed for 10 days using μCT. We assessed the correlations between joint radiological changes and clinical signs, histological changes, and serum levels of cytokines.

**Results:**

On days 5–7 after intravenous infection, bone destruction verified by μCT became evident in most of the infected joints. Radiological signs of bone destruction were dependent on the bacterial dose. The site most commonly affected by septic arthritis was the distal femur in knees. The bone destruction detected by μCT was positively correlated with histological changes in both local and hematogenous septic arthritis. The serum levels of IL-6 were significantly correlated with the severity of joint destruction.

**Conclusion:**

μCT is a sensitive method for monitoring disease progression and determining the severity of bone destruction in a mouse model of septic arthritis. IL-6 may be used as a biomarker for bone destruction in septic arthritis.

## Introduction

Septic arthritis is the most rapidly progressive joint disease. It causes severe joint inflammation followed by irreversible cartilage and/or bone destruction and subsequent permanent joint dysfunction[1, 2] The general estimated incidence of septic arthritis in industrialized countries is approximately 4–10 cases per 100,000 persons per year, with the highest rates being found in those under 15 and over 55 years old[1]. The most important risk factor for septic arthritis is pre-existing joint pathologies, in particular rheumatoid arthritis or prosthetic joint surgery[3, 4]. *Staphylococcus aureus* (*S*. *aureus*) has been reported to be the most common etiological agent[2]. The pathogenesis of septic arthritis includes a complex inflammatory response involving both the innate and adaptive immune system, as well as proinflammatory cytokines causing bone damage in the affected joints[2]. Immune-evasion strategies by *S*. *aureus* such as intracellular survival in osteoblasts[5], neutrophils[6], and endothelial cells[7] may cause the persistence of joint infection.

The study of septic arthritis in humans is hindered by the challenge to establishing the infection onset time and the difficulty in obtaining the tissue samples from the different areas of the joint[3, 4]. An optimal animal model emulating the human disease is necessary to investigate the distinct mechanisms of disease pathology in order to identify potential biological targets in the pursuit of novel therapeutics. Experimental findings from the well-established mouse model for hematogenous *S*. *aureus* septic arthritis have determined the involvement of several bacterial virulence factors in relation to host immune cell types and cytokines in the pathogenesis of this disease. This model exhibits features similar to those of human septic arthritis and provides a straightforward and rapid means of producing this pathology[8].

Over the last two decades, imaging approaches such as ultrasonography, magnetic resonance imaging (MRI) and computed tomography (CT) have made major advances in the early diagnosis and therapeutic monitoring of autoimmune joint disorders in patients[9] and in various experimental models for autoimmune joint diseases to gain a deeper understanding of disease pathophysiology[10]. Among those methods, micro CT (μCT) has been extensively used in rodent models for osteoporosis[11, 12], osteoarthritis[13], and rheumatoid arthritis[14, 15], due to the short acquisition time and instantaneous identification and quantification of disease progression. μCT was used as a supplementary method in our previous studies to determine the extent of bone destruction in mice with septic arthritis[16–18]. However, systematic descriptions of radiological changes of joints in mouse models for septic arthritis are still largely lacking.

The aim of the current study was to describe the radiological features of experimental *S*. *aureus* septic arthritis in mice. For the first time, we demonstrated that μCT is a sensitive method for monitoring disease progression and determining the severity of bone destruction in a mouse model of septic arthritis. Distal femurs in the knee joints are the most vulnerable area in septic arthritis. Importantly, IL-6 positively correlated to the severity of bone destruction verified by μCT, suggesting that IL-6 may be used as a biomarker for joint damage in septic arthritis.

## Materials and methods

### Mice

Female NMRI mice, 6–8 weeks old, were purchased from Charles River Laboratories (Sulzfeld, Germany). In total, 150 mice were used in this study. They were bred and housed in the animal facility of the Department of Rheumatology and Inflammation Research, University of Gothenburg. The mice were kept under standard conditions of temperature and light and were fed laboratory chow and water *ad libitum*. The Ethical Committee of Animal Research of Gothenburg approved the study.

### Bacterial strains and reagents

*S*. *aureus* laboratory strain Newman and clinical isolate LS-1 from mice with spontaneous outbreak of septic arthritis [19] were cultured separately on blood agar plates for 24 h, harvested, and kept frozen at -20°C in phosphate-buffered saline (PBS) containing 5% bovine serum albumin (BSA) and 10% dimethyl sulfoxide (DMSO). Before the experiments, the bacterial suspension was thawed, washed in PBS, and adjusted to the required concentration[20].

### Kinetic study of bone destruction in mice with septic arthritis

Ten NMRI mice were intravenously (i.v.) injected with *S*. *aureus* Newman (8 x10^6^ cfu/mouse). All 4 limbs were inspected for clinical signs of arthritis at day 3, day 5, day 7, and day 10 by three observers (T.J., M.N., A.A.). The mice were anesthetized with an intraperitoneal injection of 200 μl of ketamine/xylazine mixture. The affected joints with signs of septic arthritis (redness and swelling) along with both knees were then scanned *in vivo* on respective days using SkyScan 1176 micro-CT (Bruker, Antwerp, Belgium). After scanning, the mice were allowed to wake up by receiving 150–200 μl of Antisedan (Orion Pharma AB Animal Health, Sollentuna, Sweden), as the antidote and resumed the experiment until day 10.

### Experimental protocols for staphylococcal septic arthritis

To study whether bone destruction in septic arthritis are dependent on the bacterial dose, two separate experiments were performed using both *S*. *aureus* Newman and LS-1 strains. NMRI mice (n = 8–10 mice/group) were inoculated with *S*. *aureus* LS-1 (1 x 10^6^ and 1 x 10^7^ cfu/mouse) and with *S*. *aureus* Newman (2 x 10^6^ and 1.2 x 10^7^ cfu /mouse) intravenously (i.v.) into the tail vein with 0.2 ml of staphylococcal suspension in two doses. To assess the specificity of μCT analysis in determining bone erosion, joints from 5 healthy mice were collected and scanned.

The correlation between bone destruction and different parameters of staphylococcal arthritis were assessed. The mice (n = 48) were inoculated with a suboptimal arthritogenic dose of 1.1–1.7 x 10^6^ cfu of *S*. *aureus* Newman to induce variant degree of septic arthritis.

All the mice were regularly weighed and clinically examined for arthritis incidence and severity by two observers (T.J. and A.A.) blinded to the dose given to the groups. After sacrificing the mice at day 10, the kidneys were obtained for the assessment of bacterial persistence, serum samples were collected to assess the levels of cytokines and the 4 limbs were obtained for the radiological examination of bone erosion. Thereafter, the limbs from *S*. *aureus* Newman infected mice were further microscopically evaluated for the presence of synovitis and destruction of cartilage and bone.

To understand which particular joints are most often affected by septic arthritis in mice, forty-two NMRI mice were injected i.v. with the optimal septic arthritis dose (5–8 x 10^6^ cfu/mouse) of *S*. *aureus* Newman strain. All 4 limbs were collected after sacrificing the mice on day 10. μCT was carried out to determine the extent of bone destruction.

### Mouse model for local *S*. *aureus* arthritis

NMRI mice (n = 10) were inoculated intra-articularly in the knee joints with *S*. *aureus* Newman strain (1 x 10^3^ cfu/ knee). The mice were sacrificed 10 days later. Knee joints were collected for μCT scan and histological examination.

### Clinical evaluation of septic arthritis

Observers blinded to the groups visually inspected all 4 limbs of each mouse. Arthritis was defined as erythema and/or swelling of the joints. To evaluate the severity of arthritis, a clinical scoring system ranging from 0 to 3 was used for each limb (0, no inflammation; 1, mild visible swelling and/or erythema; 2, moderate swelling and/or erythema; 3, marked swelling and/or erythema). The arthritis index was constructed by adding the scores from all 4 limbs for each animal, as described previously[20]. Arthritis that involved 5 or more joints simultaneously was defined as polyarthritis.

### Bacteriologic examination

Kidneys were aseptically removed and blindly assessed by two investigators (M.N. and F.F.) for abscesses. A scoring system ranging from 0 to 3 was used (0, healthy kidneys; 1, 1 or 2 small abscesses on kidneys without structural changes; 2, more than 2 abscesses but <75% of kidney tissue involved; and 3, large abscesses with >75% of kidney tissue involved). Afterwards, the kidneys were homogenized, diluted serially in PBS, and transferred to agar plates containing 5% horse blood. Bacteria were grown for 24 h and quantified in cfu.

### Micro computed tomography

For *in vivo* kinetics study of bone destruction, the scanning was performed serially for the same animals at day 3, 5, 7, and 10 at 55 kV and 434 mA, with a 1-mm aluminum filter. The exposure time was 110 ms. The X-ray projections were obtained at 0.7° intervals with a scanning angular rotation of 180°. This resulted 35.26 of image pixel size.

For all other experiments, the joints were fixed in 4% formaldehyde for 3 days and transferred to PBS for 24 h. Afterwards, all 4 limbs were scanned and reconstructed into a three-dimensional (3D) structure with a SkyScan1176 micro-CT (Bruker, Antwerp, Belgium) with a voxel size of 35 μm. The scanning was done at 55 kV and 455 mA, with a 0.2-mm aluminum filter. The exposure time was 57 ms. The X-ray projections were obtained at 0.7° intervals with a scanning angular rotation of 180°. This also resulted 35.26 of image pixel size. The projection images were reconstructed into three-dimensional images using NRECON software (version 1.5.1; Bruker). Beam hardening correction was 30% for *in vivo* scanning and 40% for *ex vivo* scanning. After reconstruction, the 3D structures of each joint were blindly assessed by 2 observers (T.J. and F.F.) using a scoring system from 0 to 3 (0, healthy joint; 1, mild but visible bone destruction at one or more than one site; 2, moderate bone destruction at only one site; 3, moderate bone destruction at more than one site or marked bone destruction at one or more than one site).

**Fig 1:**
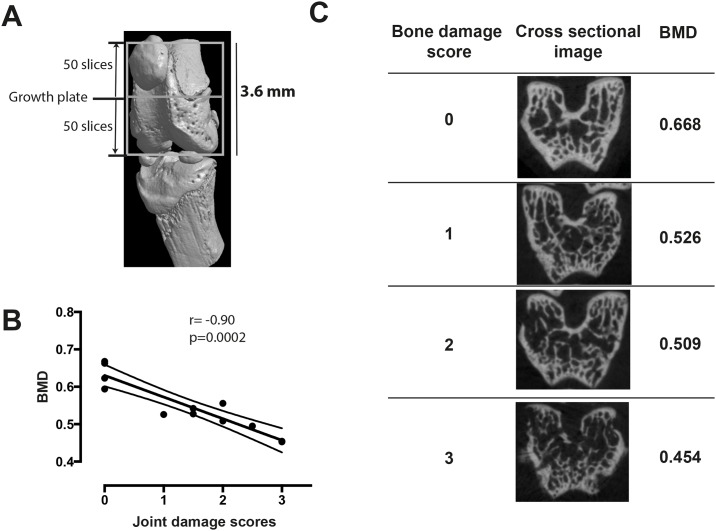
Joint damage scores verified by μCT showed good correlation with the bone morphometric parameters. The NMRI mice (n = 10) were intraarticularly injected with the *S*. *aureus* LS-1 strain (1x10^3^ cfu/mouse) in the knees for 7 days. Knee joints from those 10 mice and 2 healthy mice were analyzed by μCT scan. Joint damage was assessed by either semi-quantitative analysis using a scoring system or quantitative analysis using bone morphometric calculation. **(A)** Schematic representation showing how the section of distal femur was chosen for bone morphometric analysis; (**B**) Correlation between joint damage scores and bone mineral density; (**C**) Bone damage scores, cross-sectional images, and bone mineral density values obtained from 4 knees with various severity of joint destruction (Bone damage score varies from 0–3).

The morphometric analysis was performed for femurs from 12 knee joints including 2 healthy knees and 10 knees with various joint damage scores. A 3.6 mm section of distal femur was analyzed. The bone section was chosen from 50 slices below to 50 slices above the reference point within the growth plate (Fig 1A). The scans were reconstructed using NRecon and analyzed using the CTAn software package (Bruker-Micro-CT). Adaptive thresholding in 3D space with a radius of 3 voxels was used to segment the bone. For calculation of the bone morphometric parameters, calibration of the SkyScan CT system was performed with known density calcium hydroxyapatite phantoms (0.25 and 0.75 g/cm^3^). Thereafter, the correlation between joint damage scores and the parameters from morphometric analysis of femurs was analyzed.

### Inter- and intra- rater reliability analysis of scoring system

Five examiners (TJ, YF, AA, MLN, MM) reviewed the μCT images of 48 joints (including wrists, elbows, shoulders, ankles, knees, hips) with various severities of septic arthritis. All examiners have worked with the μCT and scoring system for at least 6 months. One examiner (TJ) read the scans twice, with an interval of 1 month between the two readings. Inter- and intra- reliability in grading bone destruction on μCT was then analyzed. Examiner agreement was categorized by kappa values as poor (<0.20), fair (0.20–0.39), moderate (0.40–0.59), good (0.60–0.79), or excellent (>0.80).

### Histopathological examination of joints

After the scanning, the joints were decalcified, embedded in paraffin, and sectioned with a microtome. Tissue sections were stained with hematoxylin and eosin. All the slides were coded and assessed in a blinded manner by two observers (M.N., and T.J.) with regard to the degree of synovitis and cartilage/bone destruction. The extent of synovitis and cartilage/bone destruction was judged as previously described [21].

### Measurement of cytokine levels

The levels of TNF-α, IFN-γ, IL-10, IL-6, IL-4 and IL-17 in serum were determined using a Cytometric Bead Array (CBA) mouse inflammation cytokine kit (BD Biosciences) and analyzed using a FacsCanto2 flow cytometer (BD Biosciences). The data were analyzed using FCAP array software (BD Biosciences).

### Measurement of bone destruction marker

The serum levels of cross-linked C-terminal telopeptides of type 1 collagen (ICTP) were determined using mouse ICTP ELISA kit according to the protocols recommended by the manufacturer (My Biosource.com) and analyzed by SoftMAx pro ELISA reader.

### Statistical analysis

The intra- and inter rater reliability analysis was assessed using Cohen and Fleiss kappa statistics by SAS (Statistical Analysis System, version 9.4). The statistical significance of differences between groups was assessed using the Mann—Whitney U test and the χ2 test. Spearman’s correlation was used to calculate correlation coefficients. The GraphPad Prism (version 6) software was used for calculations. The results are reported as the mean ± standard error of the mean (SEM) or median. A two-tailed p value <0.05 was considered statistically significant.

## Results

### Bone damage scores had a good correlation with bone morphometric parameters

The bone damage scores were semi-quantitative and obtained by subjective evaluation on 3-dimensional joint structures. To validate the scoring system, the correlation was analyzed for bone damage scores and bone morphometric parameters obtained from 12 knee joints with local *S*. *aureus* arthritis. Bone damage scores correlated well with several bone morphometric parameters (Table 1) including bone mineral density (r = -0.90, *p* = 0.0002), bone volume (r = -0.91, *p* = 0.0001), percent bone volume (bone volume/tissue volume) (r = -0.91, *p* = 0.0001), bone surface (r = -0.71, *p* = 0.01), and bone surface/volume ration (r = 0.86, *p* = 0.0007). Fig 1B illustrates the correlation between the damage scores and bone mineral density. Fig 1C shows representative cross-sectional images from 4 knees with various severity of joint destruction.

**Table 1:** Correlation between joint damage scores verified by μCT and the bone morphometric parameters.

Parameters	μCT scores	
	r	*p*
Bone mineral density	-0.90	0.0002
Tissue volume	-0.11	0.72
Bone volume	-0.91	0.0001
Bone volume/tissue volume	-0.91	0.0001
Bone surface	-0.71	0.01
Bone surface/volume ratio	0.86	0.0007
Average object area per slice	-0.52	0.086
Mean number of objects per slice	-0.50	0.099

The NMRI mice (n = 10) were intraarticularly injected with *S*. *aureus* LS-1 strain (1x10^3^ cfu/mouse) into the knees for 7 days. Knee joints from those 10 mice and 2 healthy mice were analyzed by μCT scan. Joint damage was assessed by either semi-quantitative analysis using a scoring system or quantitative analysis using bone morphometric calculation.

To further validate the scoring system, 5 independent examiners evaluated 48 joints (S1 Table). The scoring system was shown to be reliable by both inter-examiner and intra-examiner reliability analysis. There was statistically significant agreement between the first and second readings with a kappa value of 0.889 (*p*<0.0001). Moderate to good agreement was found among five examiners with a kappa value of 0.595 (p<0.001), indicating a significant agreement between the observations of all the examiners.

### Bone destruction determined by μCT became evident 7 days after infection

**Fig 2:**
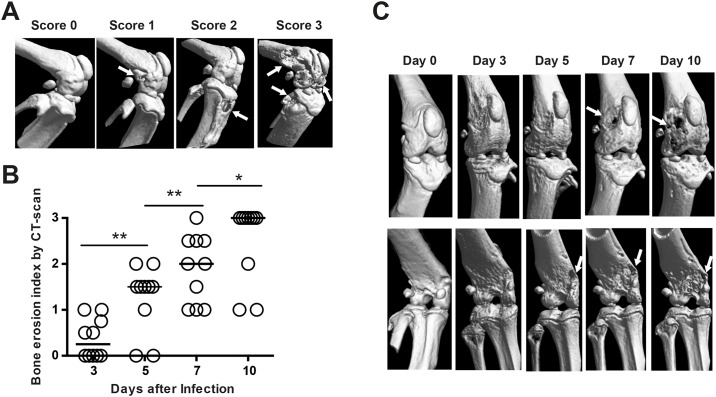
Micro-CT scan is useful to monitoring the progression of bone destruction in mice with *S*. *aureus* septic arthritis. Ten NMRI mice injected intravenously with *S*. *aureus* Newman (8 x10^6^ cfu/mouse) were serially scanned to identify bone destruction by μCT on days 0, 3, 5, 7, and 10. (**A)** Representative CT images demonstrating the scoring system for determining the extent of bone destruction in affected joints: intact knee joint (score 0), a knee joint with mild bone destruction on the distal femur (score 1; arrow), a knee joint with moderate bone erosion on the proximal tibia (score 2; arrow), and a heavily destroyed knee joint on the distal femur and proximal tibia (score 3; arrows). (**B)** Changes in bone destruction severity by μCT scan with time in affected joints. **(C)** Representative CT images in both anterior (upper panel) and posterior (lower panel) views of a knee joint showing progression of bone destruction caused by septic arthritis on different days after infection: Intact knee joint on day 0; Suspected but no clear sign of bone damages on day 3; clear signs of bone damage at the distal femur on a posterior view on day 5 (arrow); moderate bone erosion at the distal femur on both anterior and posterior views on day 7 (arrows); heavily destroyed knee joint at the distal femur and fibula on both anterior and posterior views on day 10 (arrows).

To study the development of bone destruction in mice with septic arthritis, ten NMRI mice were intravenously infected with *S*. *aureus* Newman and followed by *in vivo* CT on different days (days 3, 5, 7 and 10) after infection. The 3-dimensional joint structures were semi-quantitatively assessed by 3 observers in a blind manner (Fig 2A). The mice showing clinical signs of arthritis at day 10 also had the highest detectable bone erosion by μCT. Interestingly, on day 3, only 20% of the existing septic arthritis that later developed bone damage could be detected by μCT (Fig 2B), whereas on day 5, we were able to detect 8 out of 10 examples of bone erosion. Importantly, on day 7, bone destruction became evident in all infected joints, suggesting that μCT is sufficiently sensitive to detect most joint infections on day 7 after intravenous infection. The representative pictures (Fig 2C) demonstrate the progression of bone destruction with the passage of time. On day 0, the knee joint was completely intact and healthy. Signs of bone destruction started to appear on day 3 at the fibula, whereas structural changes also appeared on the femur. On day 5, relatively mild bone destruction became evident on both distal femur and tibia. On day 7, the distal femur and tibia were moderately infected, and heavily eroded with large bone erosion on day 10 (Fig 2C).

### Radiological signs of bone destruction detected by μCT were dependent on bacterial dose

**Fig 3:**
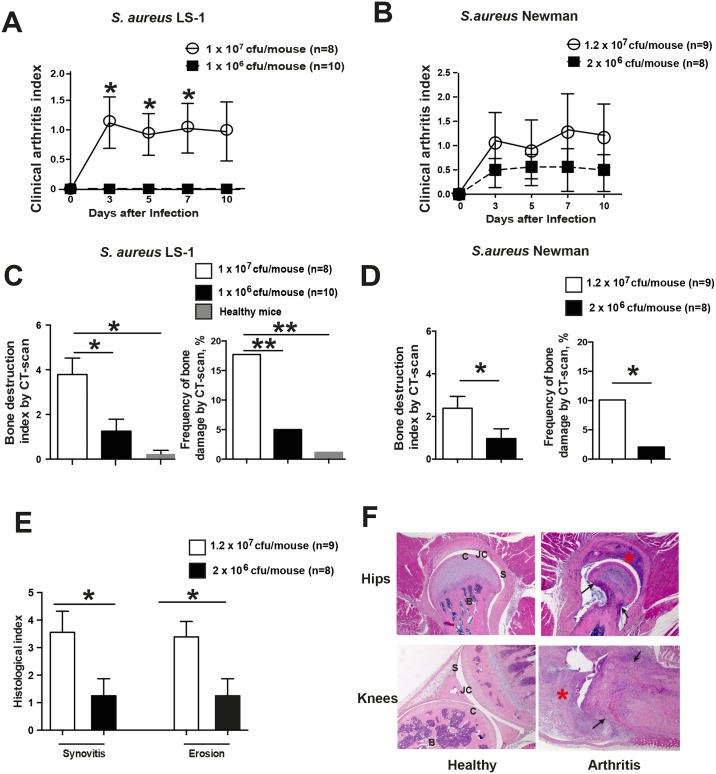
Bone destruction verified by μCT is dependent on bacterial dose in mice with septic arthritis. NMRI mice (n = 8-10/group) were inoculated intravenously with two doses of *S*. *aureus* LS-1 (1 x 10^6^–1 x 10^7^ cfu/mouse) and Newman (2 x 10^6^–1.2 x 10^7^ cfu/mouse). The severity of clinical arthritis in the mice infected with **(A)** *S*. *aureus* LS-1 and **(B)** *S*. *aureus* Newman was observed until the animals were euthanized on day 10. The bone destruction scores and frequency of bone damage from all 4 limbs of mice infected with the **(C)** LS-1 or **(D**) Newman strains were analyzed by μCT scan. **(E)** Histological evaluation of the joints from all 4 limbs 10 days after infection. **(F)** Representative micrograph of intact hip (upper left) and knee (lower left) joints as well as the heavily inflamed hip (upper right) and destroyed knee (lower right) joints from mice inoculated with *S*. *aureus* strain Newman. Original magnification ×10. The asterisks indicate heavily inflamed synovium. Arrows indicate the bone erosion. Statistical evaluations were performed using the Mann—Whitney U test. Data are presented as the mean values ± standard errors of the mean. Abbreviations: B, bone; C, cartilage; JC, joint cavity; S, synovial tissue.

A higher dose (1 x 10^7^ cfu/mouse) of the *S*. *aureus* strain LS-1 significantly increased the severity of clinical arthritis in mice compared to the lower dose (1 x 10^6^ cfu/mouse). The difference was clear already on day 3 (*p* = 0.03) after bacterial inoculation, increased over time until day 7 (*p* = 0.01) and stabilized at the end of the experiment on day 10 (*p* = 0.06) (Fig 3A). A very similar trend was also observed when the *S*. *aureus* Newman strain was used, although no statistical significance was reached (Fig 3B).

To confirm our clinical arthritis data, μCT was applied to determine whether bone destruction in joints is dependent on the bacterial dose. We found that both severity and frequency of bone erosion were significantly higher in NMRI mice infected with the higher dose of both *S*. *aureus* strains (LS-1 and Newman) compared to the corresponding lower doses. In mice infected with a higher dose of *S*. *aureus* LS-1, both the severity (*p* = 0.03) and frequency (18% vs 5%, *p* = 0.008) were significantly higher compared to the lower dose of bacteria (Fig 3C). Importantly, only 1 wrist had a suspected score of 1, and all other joints from healthy mice had a score of 0, demonstrating the good specificity of μCT analysis in determining bone destruction in septic arthritis. Although significance was not reached regarding the clinical arthritis, a significant dose-dependent pattern in both severity and frequency was observed when *S*. *aureus* Newman was used (Fig 3D).

Consistent with results from radiological examination, the extent of the histologically verified joint destruction (*p* = 0.04) as well as synovitis (*p* = 0.05) were more severe in mice receiving a higher dose of *S*. *aureus* Newman (Fig 3E) than in mice receiving a lower dose of bacteria. Fig 2F demonstrates a hip joint (top right) and a knee joint (bottom right) with septic arthritis on day 10 after infection, which is typically identified by heavily inflamed synovium and severe cartilage and bone erosion. Healthy joints, including a hip joint (top left) and a knee joint (bottom left), are characterized by a single-layer synovium without inflammatory infiltration and by intact cartilage.

Both *S*. *aureus* LS-1- and Newman-infected mice lost weight during the course of infection. Only mice receiving *S*. *aureus* LS-1 had a significantly greater weight loss (S1A and S1B Fig). In accordance with this, the only significant difference regarding the bacterial load in kidneys between high and low bacterial doses was found in mice receiving the LS-1 strain (S1C and S1D Fig).

### Knees were the most commonly affected joints in septic arthritis

**Fig 4:**
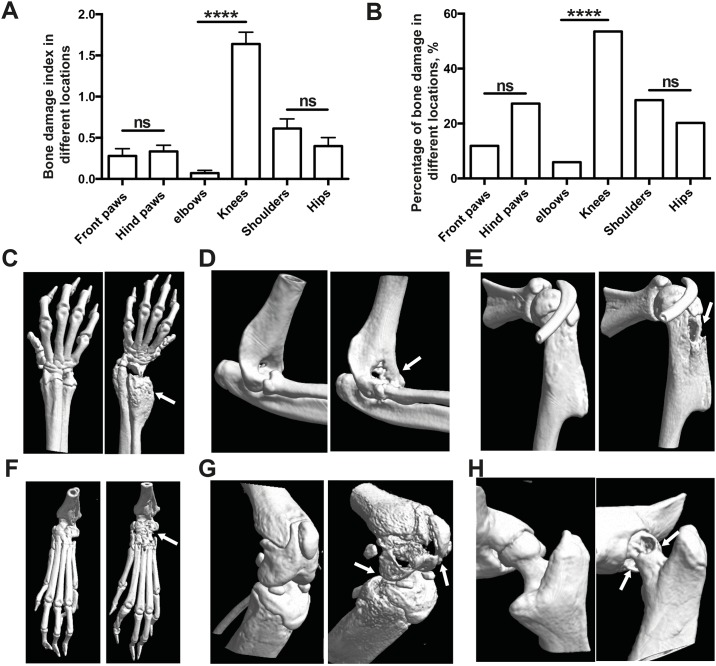
Knee joint is most often affected in *S*. *aureus* septic arthritis. NMRI mice (n = 42) were intravenously injected with *S*. *aureus* strain Newman (5–8 x 10^6^ cfu/mice) and all joints from 4 limbs were examined by μCT scan on day 10 after infection. Severity **(A)** and frequency **(B)** of bone destruction in different locations were compared. **(C-H)** Representative computed tomographic images showing both intact (Left) and heavily eroded (Right) joints, **(C)** wrist, **(D)** elbow, **(E)** shoulder, **(F)** ankle, **(G)** knees and **(H)** hips. Arrows indicate the bone erosion. Statistical evaluations were performed using the Mann—Whitney U test and Fisher exact test. Data are presented as the mean values ± standard errors of the mean.

Forty-two NMRI mice were evaluated for a more detailed subgroup analysis to investigate which particular joints were affected by septic arthritis in mice receiving optimal septic arthritis dose (5–8 x 10^6^ cfu/ mouse) of *S*. *aureus* Newman. More than half of the knee joints displayed signs of bone destruction. This was followed by 28.5% of shoulder joints, 27.2% of hind paws (including ankles and toes), and 20.2% of hip joints. In this analysis, the least frequently affected joints were the front paws (including wrists and fingers) and elbows, of which only 11.9% and 5.9% were affected, respectively. We further analyzed both the severity and frequency of bone destruction in the respective joints from upper and lower limbs (Fig 4A and 4B). Interestingly, knee joints had strikingly more frequent and severe bone damage compared to elbow joints (*p*<0.0001), whereas shoulder and hips, as well as front paws and hind paws, showed no significant difference (Fig 4B). A very similar pattern was observed when mice were infected with *S*. *aureus* clinical strain LS-1 (S2A and S2B Fig), suggesting that this finding is highly reproducible.

### Distal femur was the bone most commonly affected by septic arthritis in knees

**Fig 5:**
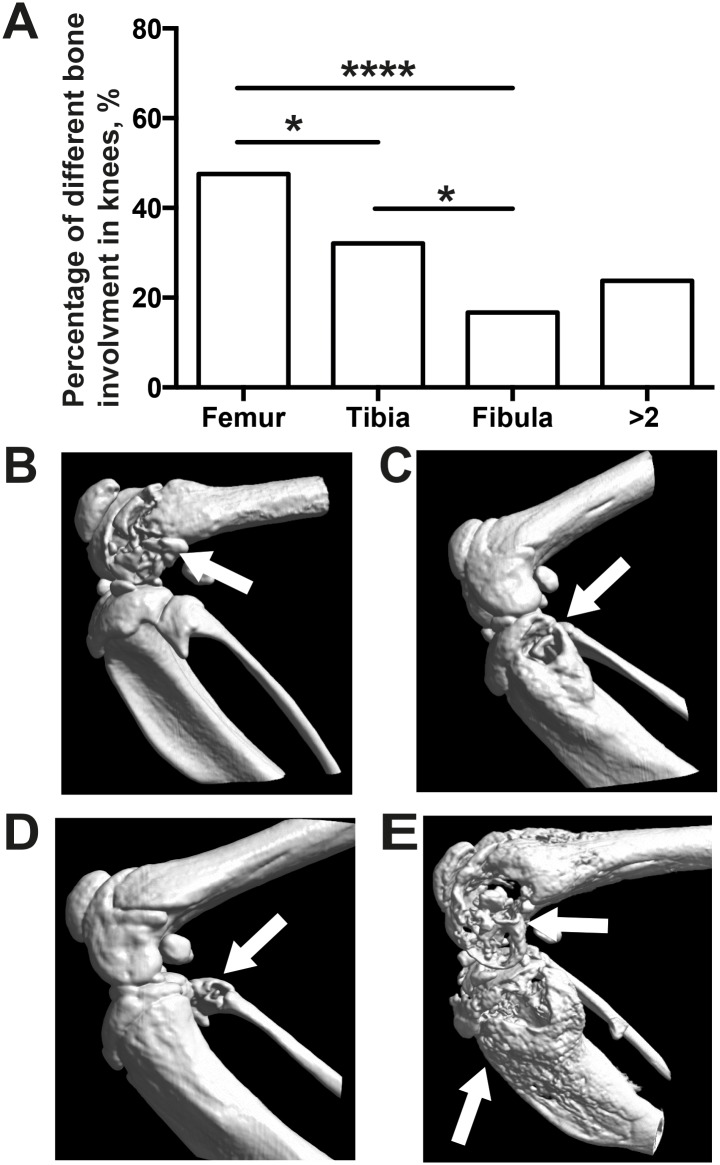
Most commonly affected site of the knee joints is the distal femur. **(A)** Percentage of different sites within the knee joints of NMRI mice infected with *S*. *aureus* Newman. **(B-E)** Computed tomography scans showing different sites of the knee affected by septic arthritis. **(B)** Heavily destroyed femur, **(C)** evident destruction on the tibia, **(D)** infected fibula, **(E)** damaged knee joints with involvement of several bones including femur, tibia and fibula.

To further evaluate which area of the knee joints are most affected during septic arthritis, we evaluated all knees from those 42 NMRI mice by μCT. The most frequently affected site in knee joints was the distal femur (47.6%), followed by proximal tibia (32.1%) and proximal fibula (16.7%) (Fig 5A). Moreover, 23.8% of knees had two or more than two bones affected.

### ICTP cannot be used as a biomarker for bone destruction in septic arthritis

Cross-linked carboxyterminal telopeptide of type I collagen (ICTP), a bone turnover marker, is considered to be useful in the early assessment of skeletal metastases in cancer patients[22, 23]. We hypothesized that ICTP may be used as a relevant bone destruction biomarker in septic arthritis. The serum levels of ICTP were compared in mice infected with different doses of *S*. *aureus* LS-1 and Newman strains on day 10. No significant difference was found between the higher and lower doses of *S*. *aureus* in both strains with regard to the bone destruction marker ICTP (supplementary S3A and S3B Fig), suggesting that ICTP is probably not a suitable biomarker for bone destruction in the late stage of septic arthritis.

### Polyarthritis is not a predictor of worse outcome of *S*. *aureus* systemic infection

**Fig 6:**
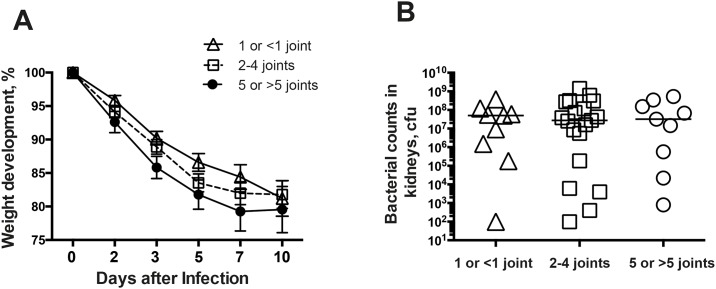
Polyarthritis is not associated with weight development and bacterial clearance in mice with *S*. *aureus* septic arthritis. Forty-two NMRI mice inoculated with the *S*. *aureus* strain Newman (5–8 x 10^6^ cfu/mice) were sacrificed 10 days after infection. Bone destruction was analyzed in all joints from 4 limbs by μCT scan. The animals were divided into 3 groups: 1) mice with 5 or more infected joints; 2) mice with 2–4 infected joints; and 3) mice with 1 or no infected joint. **(A)** Percentage changes in body weight and **(B)** persistence of *S*. *aureus* in the kidneys was recorded and compared among groups. Statistical evaluations were performed using the Mann—Whitney U test and Fisher exact test. Data are presented as the mean values ± standard errors of the mean.

Polyarthritis, defined as the mice having 5 or more infected joints, has been suggested to be indicative of poor prognosis of the disease in septic arthritis patients[4]. To study whether polyarthritis can be used as a predictor for worse outcome of *S*. *aureus* infection, 42 mice infected with *S*. *aureus* Newman were divided into 3 different groups according to the number of joints affected by septic arthritis (Group 1 with 5 or more infected joints; group 2 with 2–4 infected joints; and group 3 with 1 or no infected joint). Nine out of 42 mice (21.4%) developed polyarthritis on day 10, whereas only 4.7% of infected mice showed no sign of clinical arthritis until the end of the experiment. No significant differences were found regarding both weight loss % (Fig 6A) and kidney bacterial load (Fig 6B) among groups, indicating inadequate correlation between polyarthritis and weight loss as well as bacterial clearance in kidneys.

### Correlations between bone destruction verified by μCT and several parameters of septic arthritis

Permanent reductions in joint function due to joint destruction and deleterious contractures are known to occur in up to 50% of patients with septic arthritis[24]. There is still no biomarker available for bone destruction in septic arthritis. To this purpose, we statistically determined the correlations between radiological signs of bone destruction and serum levels of several cytokines as well as some parameters of septic arthritis in 48 NMRI mice infected with the *S*. *aureus* Newman strain (Table 2).

**Table 2:** Correlations between joint damage scores verified by micro-CT (μCT scores), kidney cfu counts and clinical signs, radiological and histological changes, and serum levels of cytokines in NMRI mice with hematogenous *S*. *aureus* septic arthritis.

Parameters	μCT scores		Kidney cfu counts	
	r	*p*	r	*p*
Clinical arthritis scores	0.28	0.05		NS
Weight development, %	-0.42	0.0028	-0.362	0.0115
Kidney cfu counts		NS		_
Histo synovitis	0.54	<0.0001		NS
Histo erosion	0.52	0.0001	_	NS
μCT scores	_	_		NS
TNF-alpha		NS	0.67	<0.0001
IFN-gamma		NS		NS
IL-10		NS		NS
IL-6	0.41	0.0042	0.36	0.0154
IL-4		NS		NS
IL-17		NS		NS

The NMRI mice (n = 31) were intravenously inoculated with *S*. *aureus* Newman strain (1.1–1.7x10^6^ cfu/mouse). The clinical signs including clinical arthritis scores, weight development %, and kidney cfu counts were registered on day 10 after infection. All joints from 4 limbs were analyzed by μCT and histological examination. Serum levels of cytokines including TNF-alpha, IFN-gamma, IL-10, IL-6, IL-4, and IL17 were analyzed.

Bone destruction by μCT analysis was positively correlated with histological changes (p = <0.0001, r = 0.54), suggesting the high accuracy and sensitivity of μCT in detecting septic arthritis. It also showed a relatively good correlation with weight loss % (p = 0.0028, r = -0.42) and serum level of inflammatory cytokine IL-6 (p = 0.0042, r = 0.41). This suggests that IL-6 may be used as a biomarker for bone erosion in septic arthritis. A significant correlation was also found between clinical arthritis score and bone destruction determined by μCT (p = 0.05, r = 0.28).

In the case of kidney bacterial counts, the best correlation was found with serum levels of the proinflammatory cytokine TNF-α (p = <0.0001, r = 0.67). Significant correlations were also found with weight loss % (p = 0.0115, r = -0.36) and serum levels of IL-6 (p = 0.0154, r = 0.35).

### Radiological and histological signs correlated well in a local infection model of septic arthritis

NMRI mice infected with the *S*. *aureus* Newman strain (1 x 10^3^ cfu/knee) in the knee joints were evaluated for bone destruction by μCT after sacrifice on day 10. Thereafter, histological analysis was also carried out. A significant correlation was found between the radiologically determined bone destruction by μCT and histologically determined signs of erosion (*p* = 0.0088, r = 0.56, Table 3) as well as signs of synovitis (*p* = 0.0089, r = 0.56).

**Table 3:** Correlation between joint damage scores verified by μCT (μCT scores) and histological scores including severity of synovitis and bone erosion in NMRI mice with local *S*. *aureus* arthritis.

Histological scores	μCT scores	
	r	*p*
**Synovitis**	0.5685	0.0089
**Erosion**	0.5693	0.0088

NMRI mice (n = 10) were inoculated intra-articularly in the knee joints with *S*. *aureus* Newman strain (1x10^3^ cfu/knee). The mice were sacrificed 10 days later. Knee joints were collected for μCT scan and histological examination.

## Discussion

μCT offers the unique opportunity to visualize three-dimensional micro bone architectural changes occurring in the various types of arthritis models. Since extremely aggressive and rapid bone destruction is one of the major hallmarks of septic arthritis[1, 2], we hypothesized that μCT could be an excellent tool for the assessment of bone damage in our murine model of septic arthritis. In this study, we systematically studied the radiological features of *S*. *aureus* septic arthritis (both hematogenous and local) in mice by μCT. Our data suggest that μCT is a useful supplementary method for clinical assessment and bacteriological analysis in a mouse model of septic arthritis.

Obviously, quantitative bone morphometric analysis is a more sensitive and objective method compared with the scoring system. However, since almost all joints (both large and small joints) can be involved in hematogenous *S*. *aureus* septic arthritis, it is necessary to analyze all joints in order to measure the severity of bone destruction in each individual, which is extremely time-consuming. Therefore, we concluded that although quantitative analysis is a more sensitive method, the scoring system is probably a more pragmatic approach for determining the severity of bone erosion in mice with hematogenous septic arthritis. However, in the case of local *S*. *aureus* arthritis (i.e. local injection of *S*. *aureus*), quantitative analysis may be a very useful tool and better method to analyzing the bone destruction.

As various *S*. *aureus* strains differ in several virulence determinants, the radiological pattern of bone destruction caused by different strains may exhibit some variations. In the present study, both a clinical strain (LS-1) and laboratory strain (Newman) were used, which differed in the expression of several virulence factors. For instance, Newman strain is known to express staphylococcal enterotoxin-A (SEA) but lacks the *tst* gene, which produces toxic shock syndrome toxin-1 (TSST-1)[25]. In contrast, the LS-1 strain has been shown to produce large amounts of TSST-1[26]. Despite those differences between the two strains, the clinical features and radiological changes in mice with septic arthritis induced by these two strains were very similar, suggesting that radiological evaluation of bone damage by μCT is stable and consistent in septic arthritis elicited by different *S*. *aureu*s strains.

To determine whether the appearance of radiological signs was dependent on the bacterial dose, bone erosion severity and frequency were compared using μCT following the administration of a standard low dose of *S*. *aureus* inoculum (10^6^ CFU/mouse) versus a 1-log-higher dose (10^7^ CFU/mouse). Despite the fact that no significant difference was observed in the clinical evaluation of arthritis between different doses, significant differences were observed in both the severity and frequency of bone damage by μCT, indicating good sensitivity of this method for the detection of bone damage. In addition, the μCT data also correlated well with histological evaluations that are the gold standard for the determination of disease onset and progression in our model, further supporting the potential of μCT for disease evaluation. These observations suggest that μCT can be used as an alternative non-invasive technique for disease index assessment and to quantify bone damage in a septic arthritis mouse model.

With more than 20 years of experience with our unique septic arthritis model, we noticed that a major limitation of the clinical assessment of septic arthritis in mice: clinical arthritis scoring is usually impossible when assessing deep joints, including the knees, elbows, hips, and shoulders, since redness and swelling in those joints is undetectable by clinical observation. Our data demonstrated that μCT could easily detect these deeper joints that are extensively involved during septic arthritis, hence overcoming the limitation of the clinical arthritis assessment.

The most frequently involved joints in patients with septic arthritis are the knees, followed by hips, shoulders, wrists and ankles[24], which is in full agreement with current data in our animal model. This is another strong piece of evidence supporting the idea that our mouse model for septic arthritis closely emulates the human disease. It has been repeatedly observed that the larger joints in the weight-bearing lower limbs are more commonly involved in septic arthritis compared to the upper extremities[27]. More than 50% of knees affected by septic arthritis compared to the 5.9% of elbow joints in our study demonstrate a similar pattern. Strikingly, a higher involvement of knees with septic arthritis than elbows was also observed in our previous studies[16]. Previously, a proposed explanation for this pattern was that generally it is easier to aspirate synovial fluid from the large joints of the lower limbs, such as the knee, compared with joints of the upper extremities during routine examination of septic arthritis patients. Our data suggest that the abovementioned explanation may be imprecise. We hypothesize that the differences in joint anatomy or in expression of host adhesion molecules that bind bacteria may be the cause of such variation. However, future studies are largely needed to elucidate the underlying mechanism.

Hitherto, knees have been most often involved in *S*. *aureus*-induced septic arthritis, but it was largely unknown which area of knees was most susceptible to bacterial infection. In the present study, the distal femur was demonstrated to be most frequently eroded compared to tibia and fibula, suggesting that the distal femur is the most vulnerable region during the course of septic arthritis. Osteosarcoma, a primary bone tumor, is also most commonly found in the distal femur, which is an anatomic site associated with abundant blood supply[28]. We hypothesize that those two distinct joint diseases may have some similarities in their early stages of disease development.

Early diagnosis and prompt treatment with appropriate antibiotics are crucial determinants for better prognosis in septic arthritis. Indeed, the discovery of new diagnostic biomarkers for septic arthritis would enable early intervention and intensive treatment of the disease, which is otherwise delayed, resulting in permanent joint and bone damage. So far, there is no evidence of a reliable bone marker to detect early bone destruction in septic arthritis patients. Serum ICTP has been shown to be a valuable index of bone turnover in several pathological situations, including bone metastases of breast[29], prostate[22], and lung[30] cancer cells, and multiple myeloma[23]. However, in the present study, no significant association was observed between serum ICTP levels and bone damage on day 10 after infection, demonstrating it to be an inadequate marker in the late stage of septic arthritis.

Increased levels of cytokines such as IL-l, IL-6 and TNF have been interpreted as indicators of the inflammatory state. It is unlikely that these cytokines could serve as “biomarkers” in inflammatory disease, as they are linked to general disease biological processes and hence not specifically associated with a particular disease. Additionally, a lack of correlation is often observed between cytokine levels (in serum and/or plasma) and clinical endpoints. Surprisingly, in our study, a positive correlation of IL-6 level with the severity of bone destruction verified by μCT suggests that IL-6 may be a potential biomarker of the extent of bone damage in septic arthritis. Indeed, despite not being disease specific, IL-6 has been shown to be more sensitive than other serum cytokines for the prediction of therapeutic response of rheumatoid arthritis patients[31]. Bacterial load in the kidneys, one of the most important parameters for *S*. *aureus* systemic infections, reflects the strength of the host immune system against invaders. The excellent positive correlation between bacterial counts in the kidneys and TNF levels in the serum strongly indicates the potent role of TNF in *S*. *aureus* systemic infections, which is in agreement with our previous studies[17, 20].

Polyarthritis is known to be indicative of poor prognosis in septic arthritis and associated with a higher mortality[4]. Surprisingly, our data demonstrate that mice with polyarthritis had similar weight gain and almost identical kidney CFU counts compared with mice with arthritis in 1 or less than 1 joint, suggesting that although bacteremia initiates hematogenous septic arthritis, the severity of joint infections is independent of the disease parameters of sepsis, such as weight loss and kidney bacterial load.

There are many advantages of using μCT in our mouse model for septic arthritis. First, as mentioned before, it can easily detect deep joints including knees, hips and shoulders, which cannot be assessed by clinical analysis. Second, this non-invasive imaging technology enables *in vivo* scanning to follow up the joint morphological changes occurring in the same animals at different time intervals instead of sacrificing groups of animals at given time points. Third, the technique allows the visualization of the external and inner area of the same joints without altering the specimen, making it useful for later histological analyses[15]. Importantly, the excellent correlation between μCT analysis and histological evaluation of septic arthritis demonstrate the accuracy and sensitivity of μCT to identify the joints with septic arthritis, at least in the late phase of the disease.

Clearly, μCT also has some limitations. Despite the fact that major radiological changes in bone destruction became visible in some joints (20%) already on day 3 after infection, the earliest time-point to definitely identify bone erosion caused by septic arthritis was relatively late (7 days post infection or later), suggesting that μCT imaging is not so useful for early diagnostic of septic arthritis in clinical practice. The reason may be that μCT is not sufficiently sensitive to detect joint effusion, soft tissue swelling, and para-articular abscesses, which usually occur several days before bone damage. For early diagnosis, the magnetic resonance imaging is known to be more sensitive with high accuracy[32]. A standard scan of all joints of one mouse generates an image-volume that is several GB in size[33]. Computers require multiple, high-end graphic cards as well as large amounts of memory to reconstruct the generated images into 3D volumes. The management and analyses of such large volumes of data can sometimes be problematic[33].

## Conclusion

In summary, for the first time we systematically studied the radiological features of bone damage in mice with septic arthritis using μCT. As in the human disease, the most commonly affected joints were the knees, and the distal femur was the most susceptible site. The bone destruction detected by μCT analysis was positively correlated with histological changes and clinical signs of arthritis. Interestingly, serum levels of IL-6 were significantly correlated with the severity of bone destruction in septic arthritis. Our data strongly suggest that μCT is a useful non-invasive technique for evaluating the extent of joint destruction in a mouse model of septic arthritis.

## Supporting information

S1 Table Bone damage scores obtained by intra- and inter- raters from μCT analysis in 48 joints. (PDF)

S1 Fig The weight development and bacterial load in kidneys in mice with *S*. *aureus* septic arthritis. NMRI mice (n = 8–10) inoculated intravenously with two doses of *S*. *aurues* LS-1 (1x 10^6^–1x 10^7^ cfu/mouse) and *S*. *aurues* Newman (2x 10^6^–1.2 x 10^7^ cfu/mouse) were sacrificed on day 10 after infection. **(A-B)** Percentage changes in body weight registered from day 0 in mice infected with **(A)** *S*. *aureus* LS-1 and **(B)** *S*. *aureus* Newman. **(C-D)** Persistence of bacterial strains **(C)** *S*. *aureus* LS-1 and **(D)** *S*. *aureus* Newman in kidneys of NMRI mice. Mean±SEM. * p<0.05. ** p<0.01. Mann-Whitney U test.
(TIF)

S2 Fig Knee joint is the most often affected in septic arthritis induced by *S*. *aureus* LS-1. NMRI mice (n = 8–10) were intravenously injected with *S*. *aureus* LS-1 (1x10^6^–1x 10^7^ cfu/mouse) and all joints from 4 limbs were examined by μCT scan on day 10 after infection. Severity **(A)** and frequency **(B)** of bone destruction in different locations were compared. ns = not significant; Mann-Whitney test *U* test or Fisher’s exact test.
(TIF)

S3 Fig Serum levels of cross-linked carboxyterminal telopeptide of type I collagen (ICTP) are not associated with extent of bone destruction in septic arthritis. NMRI mice (n = 8–10) were inoculated intravenously with two doses of *S*. *aurues* LS-1 (1x 10^6^ -1x 10^7^ cfu/mouse) and *S*. *aurues* Newman (2x 10^6^–1.2 x 10^7^ cfu/mouse) were sacrificed on day 10 after infection. Blood was collected and serum levels of ICTP were determined. Mean±SEM; ns = not significant; Mann-Whitney U test.
(TIF)
