# Supplementary material for: Radiological features of experimental staphylococcal septic arthritis by micro computed tomography scan
Source: PLoS One. 2017 Feb 2;12(2):e0171222. doi: 10.1371/journal.pone.0171222 (PMC5289588; doi:10.1371/journal.pone.0171222)
Supplement: S1 Table — (PDF) [file pone.0171222.s001.pdf]

	Joint no.	Rater1	Rater1 (1 month later)	Rater2	Rater 3	Rater4	Rater5
**Front paws**	1	3	3	3	3	3	3
	2	3	3	3	3	3	3
	3	2	2	3	3	2	3
	4	1	1	1	1	1	1
	5	3	3	3	3	3	3
	6	1	0	0	0	1	1
	7	3	3	3	2	3	3
	8	0	0	0	0	0	0
**Hind paws**	9	1	1	0	1	1	1
	10	2	2	2	3	2	2
	11	2	2	2	1	3	2
	12	1	1	2	1	3	1
	13	0	0	1	1	1	1
	14	3	3	2	2	3	3
	15	3	3	2	3	3	3
	16	0	0	0	0	0	0
**Elbows**	17	1	2	2	2	2	2
	18	1	1	1	1	1	1
	19	1	1	1	1	1	1
	20	3	3	3	3	3	2
	21	0	0	0	0	0	0
**Knees**	22	2	2	3	3	3	3
	23	3	3	3	3	3	3
	24	3	3	3	2	3	3
	25	2	2	1	2	2	1
	26	2	2	1	2	3	3
	27	1	1	1	1	2	1
	28	3	3	3	3	3	3
	29	3	3	3	3	3	3
	30	2	2	2	2	3	2
	31	0	0	0	0	0	0
**Shoulders**	32	3	3	3	3	3	3
	33	2	2	2	2	3	2
	34	3	3	2	2	2	2
	35	3	3	3	3	3	3
	36	2	2	2	2	2	2
	37	2	2	2	2	3	3
	38	1	1	1	0	1	1
	39	1	2	2	2	3	2
	40	0	0	0	0	0	0
**Hips**	41	3	3	3	3	3	3
	42	2	2	2	2	3	2
	43	2	2	2	2	2	3
	44	2	3	3	3	3	3
	45	2	2	3	2	3	2
	46	1	1	1	2	2	1
	47	3	3	3	3	3	3
	48	0	0	0	0	0	0
