# Supplementary figures and images for: Radiological features of experimental staphylococcal septic arthritis by micro computed tomography scan
Source: PLoS One. 2017 Feb 2;12(2):e0171222. doi: 10.1371/journal.pone.0171222 (PMC5289588; doi:10.1371/journal.pone.0171222)

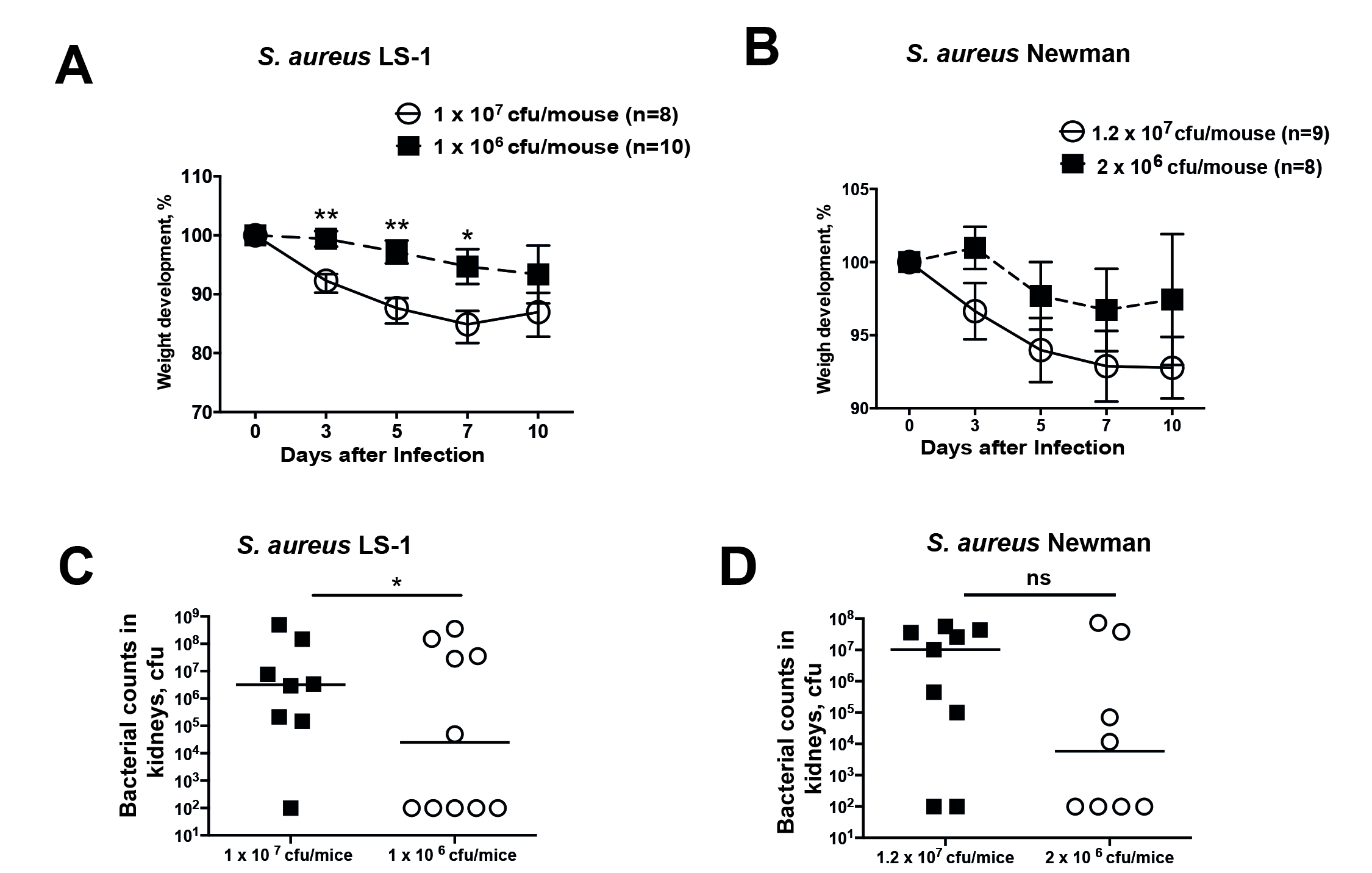

Supplement: S1 Fig — NMRI mice (n = 8–10) inoculated intravenously with two doses of S. aurues LS-1 (1x 10^6^–1x 10^7^ cfu/mouse) and S. aurues Newman (2x 10^6^–1.2 x 10^7^ cfu/mouse) were sacrificed on day 10 after infection. (A-B) Percentage changes in body weight registered from day 0 in mice infected with (A) S. aureus LS-1 and (B) S. aureus Newman. (C-D) Persistence of bacterial strains (C) S. aureus LS-1 and (D) S. aureus Newman in kidneys of NMRI mice. Mean±SEM. * p<0.05. ** p<0.01. Mann-Whitney U test. (TIF) [file pone.0171222.s002.tif]

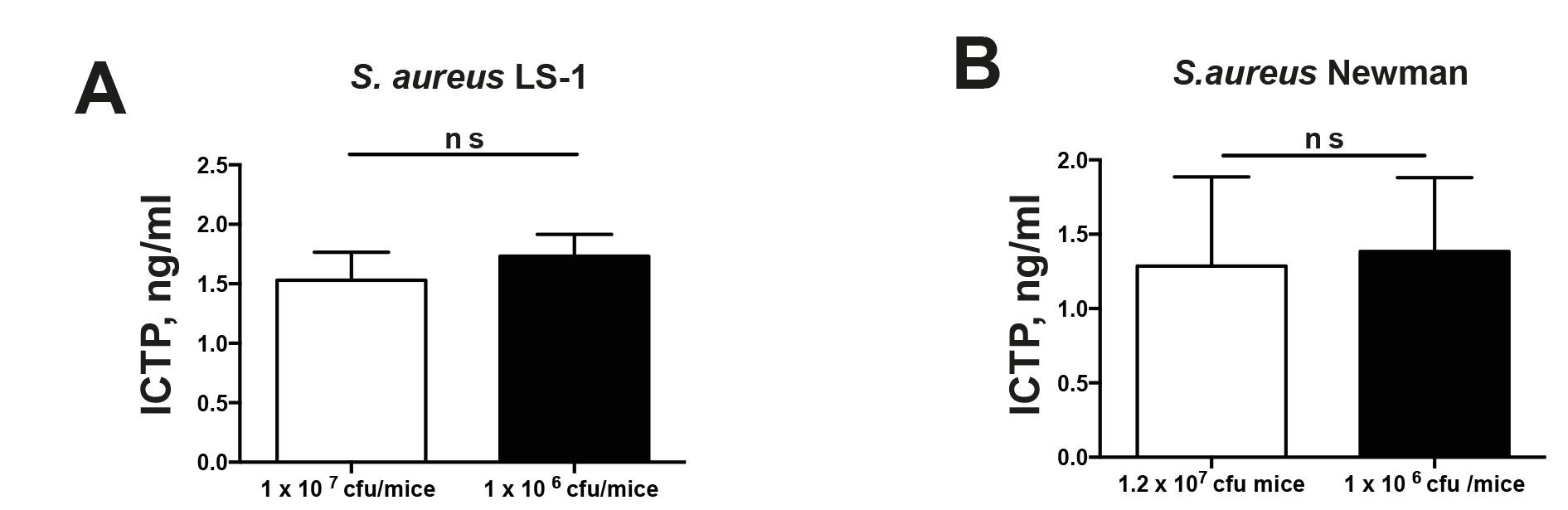

Supplement: S2 Fig — NMRI mice (n = 8–10) were intravenously injected with S. aureus LS-1 (1x10^6^–1x 10^7^ cfu/mouse) and all joints from 4 limbs were examined by μCT scan on day 10 after infection. Severity (A) and frequency (B) of bone destruction in different locations were compared. ns = not significant; Mann-Whitney test U test or Fisher’s exact test. (TIF) [file pone.0171222.s003.tif]

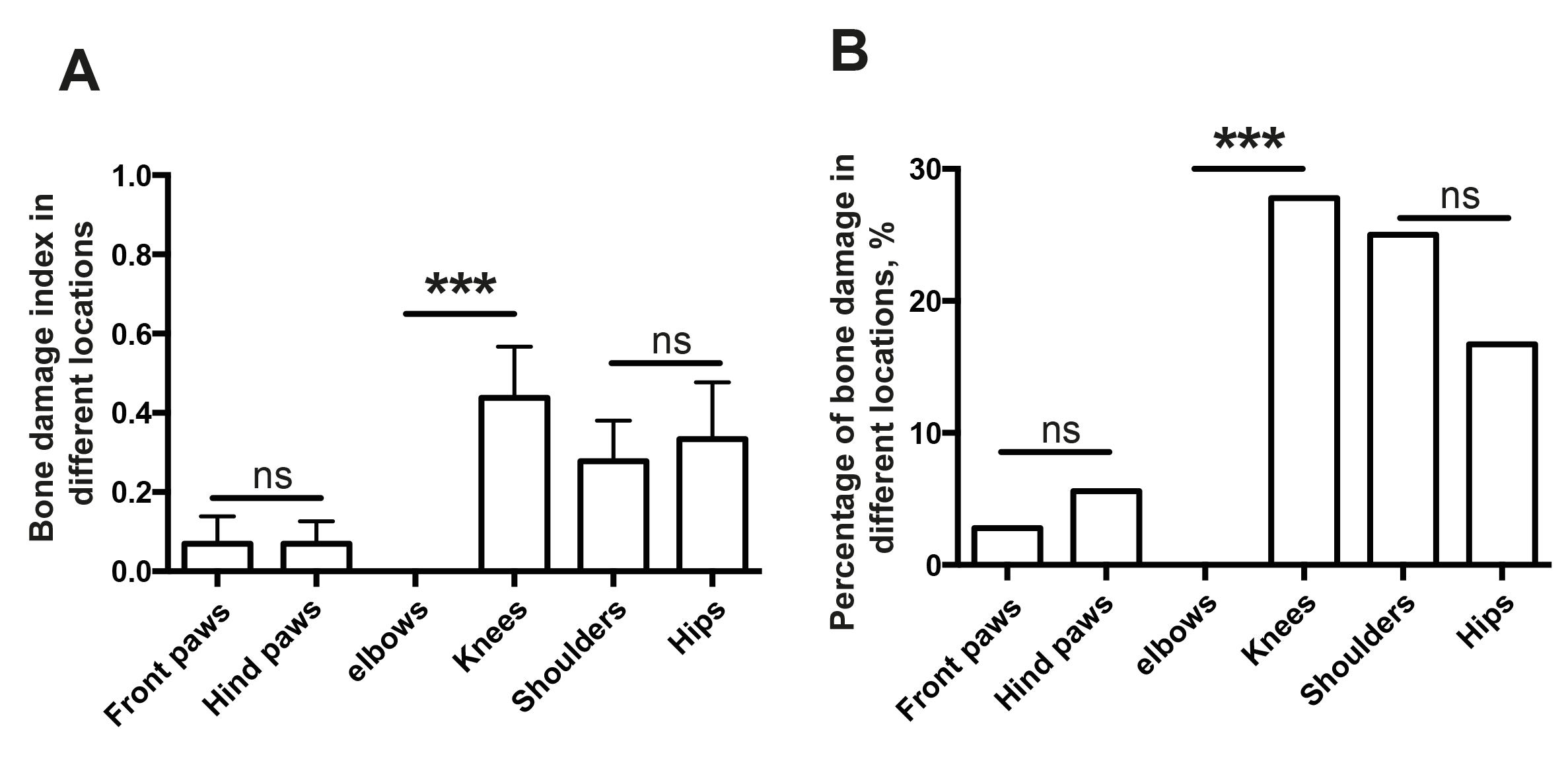

Supplement: S3 Fig — NMRI mice (n = 8–10) were inoculated intravenously with two doses of S. aurues LS-1 (1x 10^6^ -1x 10^7^ cfu/mouse) and S. aurues Newman (2x 10^6^–1.2 x 10^7^ cfu/mouse) were sacrificed on day 10 after infection. Blood was collected and serum levels of ICTP were determined. Mean±SEM; ns = not significant; Mann-Whitney U test. (TIF) [file pone.0171222.s004.tif]
